# 830. A Bovine Dermal Collagen Matrix for Deep Partial-thickness Burns: A Case Series

**DOI:** 10.1093/jbcr/irag033.269

**Published:** 2026-04-06

**Authors:** Michael R Young, Patrick John Kennedy, Olivia Duru, Nidhi D Aravapalli, Laura K Pezzopane, Beth McGuire, Ariel Rodgers, Nicole P Bernal, John H Loftus

**Affiliations:** The Ohio State Comprehensive Burn Center, Columbus, OH; The Ohio State Comprehensive Burn Center, Columbus, OH; The Ohio State Comprehensive Burn Center, Columbus, OH; The Ohio State Comprehensive Burn Center, Columbus, OH; The Ohio State Comprehensive Burn Center, Columbus, OH; The Ohio State Comprehensive Burn Center, Columbus, OH; The Ohio State Comprehensive Burn Center, Columbus, OH; The Ohio State Comprehensive Burn Center, Columbus, OH; The Ohio State Comprehensive Burn Center, Columbus, OH

## Abstract

**Introduction:**

Dermal matrices have become an integral part of modern burn care due to their ability to reduce pain, enhance cosmesis, and restore function in full-thickness wounds. In the context of deep partial-thickness wounds, dermal matrices can serve as an advanced dressing option to support re-epithelialization, potentially eliminating the need for traditional autografting. A bovine dermal collagen matrix (BDCM) comprised of crosslinked, type I and type III collagen has demonstrated effectiveness in supporting rapid cellular infiltration and vascular tissue formation in porcine full-thickness wounds preclinically as well as facilitating early autograft readiness in two recent clinical cases. Building on its utility in full-thickness wounds, our institution was the first to evaluate the clinical efficacy of BDCM as an advanced dressing in deep partial-thickness burns.

**Methods:**

A retrospective chart review was conducted from March to May 2025 to evaluate the safety and effectiveness of BDCM in 3 patients with deep partial-thickness burns. Outcomes assessed included matrix integration, time to re-epithelialization, extent of repigmentation in treated areas, and pain assessment.

**Results:**

In case 1, an 18-year-old male with a 3% total body surface area (TBSA) deep partial-thickness grease burn to the right lower leg, ankle, and foot was treated with BDCM in combination with a skin cell suspension autograft (SCSA). He was discharged on post-operative day (POD) 1 with full ambulation and a night splint. On POD 6, the BDCM had fully integrated, with epidermal budding and minimal open areas; the patient reported no pain. On POD 13, 100% re-epithelialization was noted, and melanin pigment had begun to return.

In case 2, a 51-year-old male had a 0.5% TBSA deep partial-thickness chemical burn to bilateral hands and wrists. On POD 7, the BDCM had fully integrated with one small open area on the fifth digit. Complete (100%) re-epithelialization was achieved by POD 20.

In case 3, a 33-year-old male with an 8% TBSA deep partial-thickness scald burn to bilateral lower extremities was treated with BDCM and SCSA. On POD 8, the BDCM had mostly incorporated with 95% re-epithelialization. By POD 15, early repigmentation was noted.

**Conclusions:**

These cases demonstrate the safety and efficacy of the BDCM in supporting healing of deep partial-thickness burns. Importantly, the BDCM supported re-epithelialization and repigmentation, thus offering a more diverse treatment option within burn care for deep partial-thickness injuries. Additionally, patients experienced reduced pain post-application and simplified aftercare, further highlighting the clinical utility of the BDCM.

**Applicability of Research to Practice:**

BDCM is a safe and viable advanced dressing option for healing deep partial-thickness burns.

**Funding for the study:**

N/A.

